# Role of IGF2BPs in head and neck squamous cell carcinoma

**DOI:** 10.3389/fonc.2022.1003808

**Published:** 2022-09-27

**Authors:** Kainan Wu, Fen Chang, Wenming Li, Tongdong Su, Dapeng Lei

**Affiliations:** ^1^ Key Laboratory of Otolaryngology, NHFPC (Shandong University), Shandong, China; ^2^ Department of Otorhinolaryngology, Qilu Hospital, Shandong University, Shandong, China

**Keywords:** IGF2BPs, head and neck squamous cell carcinoma, RNA binding proteins (RBPs), molecule mechanism, Treatment

## Abstract

IGF2BPs belongs to a family of conserved RNA-bound oncoembryonic proteins that play a crucial part in various aspects of cell function, such as cell migration, morphology, metabolism, proliferation and differentiation. Recent studies have shown that IGF2BPs play a role as a member of m6A reader. m6A is the most abundant modification in RNA epigenetics, which is closely related to a family of RNA-binding proteins. These proteins are fell into three categories—writers, readers and erasers. In the present study, IGF2BPs play an important role in tumor metabolism, especially in head and neck squamous cell carcinoma (HNSCC) metabolism. In this paper, the basic structure of IGF2BPs, its role in the development of HNSCC, molecular mechanism, research progress and research prospect of IGF2BPs in HNSCC are reviewed, which will providing new ideas for further study of IGF2BPs.

## Structure

**Figure 1:**
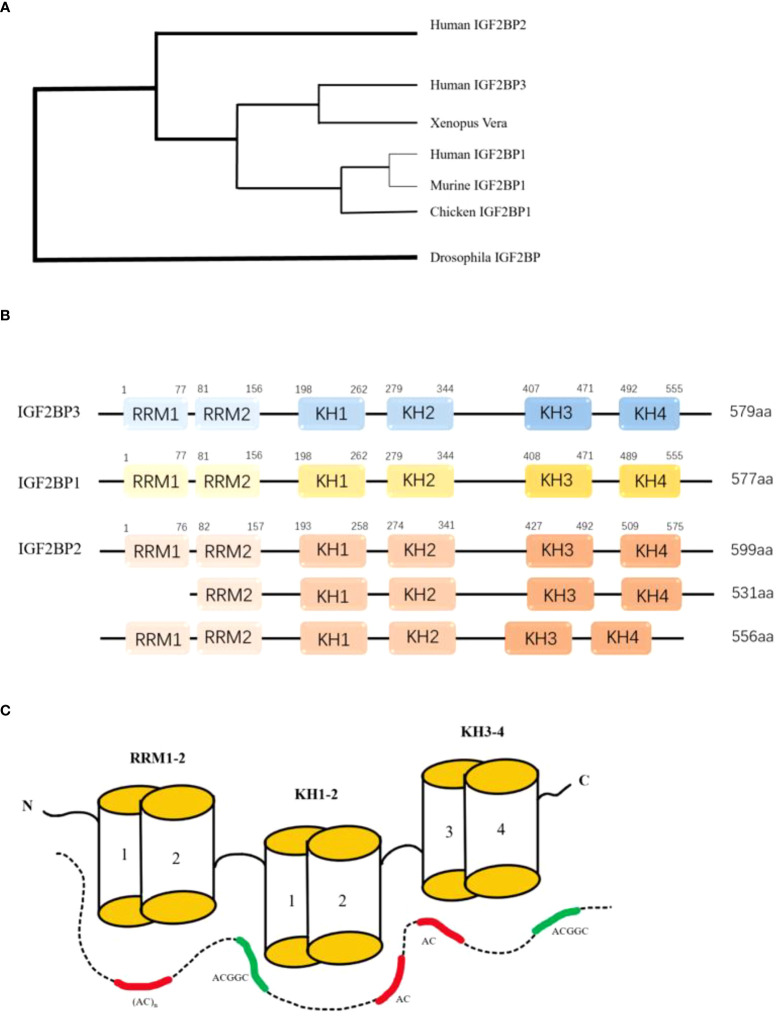
**(A)** The development tree of IMPs' homologous gene. **(B)** The six RNA binding domains of IGF2BPs: two RNA recognition regions at the N-terminal and four K homology regions at the C-terminal. All four KH domains contribute to RNA binding, ribonucleoprotein particle formation, and cell localization. **(C)**Model for RNA recognition by IGF2BPS. The combinations of the GGC-core elements with CA-rich motifs are shown for full-length IGF2BPs and the KH-containing derivatives. RCM: RNA recognition motif. KH:hnRNP-K homology domain.

IGF2BPs (Insulin-like Growth Factor 2 mRNA-binding Protein), are also known as IMP, CRD-BP, VICKZ, ZBP, Vg1RBP/Vera, or KOC. At present, there are three proteins in IGF2BPs’ family, namely IGF2BP1, IGF2BP2 and IGF2BP3, which are located on human chromosome 17q21.32 (1) 、3q27.2 (2) 、7p15.3 (3) respectively. The awkward naming of the IGF2BPs’ family reflects the diversity of the development of IGF2BPs’ homologous gene (4) (**Figure 1A**).

Typical structures of the three IGF2BPs proteins are strikingly similar in domain sequence and spacing (**Figure 1B**), with molecular weights ranging from 58 to 66 kDa. The consistency of amino acid sequences between these three proteins was over 56%, with IGF2BP1 and IGF2BP3 agreeing even more with each other at 73% (5). There is also a high degree of similarity within the protein domain. Tim Schneider et al. combined single-domain analytical Selex-seq, Motif-Spacing analysis, *in vivo* iCLIP, functional verification analysis, and structural biology to determine the RNA binding specificity and RNP topology of IGF2BPs. Including 6 RNA-binding Domains (RBDs) and a cluster of RNA elements rich in CA and GGC cores (6) (**Figure 1C**). These similarities suggest that the proteins have the same biochemical function. IGF2BPs have a special structure consisting of six RNA binding domains: two RNA recognition regions at the N-terminal and four K-homology regions at the C-terminal. These domains include: RRM1, RRM2, KH1, KH2, KH3, KH4. All four KH domains contribute to RNA binding, ribonucleoprotein (RNP) particle formation and cell localization (3). *In vitro* studies have shown that binding RNA is promoted primarily through the KH domain, although the RRM domain may contribute to the stabilization of the IGF2BP-RNA complex (7). Both RRM domains adopt a typical RRM topology, that is, two α-helices are filled on an antiparallel four-strand β slice. The spatial orientation of RRM1 to RRM2 is unique. In RNA-bound IGF2BPs-RRM12 complex, only RRM1 is involved in RNA binding and recognition of dinucleotide sequences (8). The ability of IGF2BPs to bind easily to RNA also determines its subcellular localization, and IGF2BPs are mainly concentrated in cytoplasmic particles, especially in the perinuclear region (7). However, these proteins have also been observed in the nucleus, suggesting that the subcellular classification of this protein family is essentially regulated by their association with specific RNA substrates (9).

## Discovery process of IGF2BPs

In 1972, Daughaday WH et al. proposed the term “somatomedin” to refer to those GH-dependent growth factors in serum (10). In 1976, E Rinderknecht and R E Humbel discovered that human serum contained a substance with insulin-like activity that was not inhibited by insulin antibodies (NSILA). Two forms of NSILA (NSILA I and II) have been isolated from human serum. NSILA I and Il are two forms of an insulin hormone that promotes cell and tissue growth (11). In 1987, The scientific community reached a consensus to classify the two substances into one (12). IGF2BPs are well expressed throughout development and in reproductive tissues (with high proliferation needs). The expression patterns of IGF2BP1 and IGF2BP3 can be described as “cancerous embryos”, as they are largely absent in adult tissues but are severely upregulated in various tumors and tumor-derived cells (13). Compared with IGF2BP1 and IGF2BP3, IGF2BP2 has been considered as a candidate gene for type 2 diabetes mellitus (T2D) (14).

**Figure 2:**
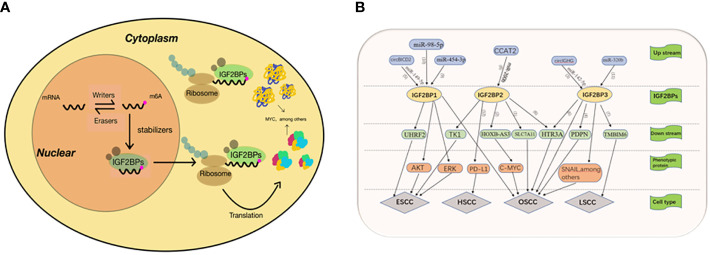
**(A)** 1GF23Ps-mediated regulation of m6A modified mRNA. The mRNA is methylated by a methyltransferase complex, which is composed of METTL3, METTE14 and WTAP. The original RNA with m6A modification was preferentially recognized by IGF2BPS. IGF2BPs protects target mRNAs from degradation by recruiting mRNA stabilizers, such as HuR and Matri3, while promoting mRNA transfer to cytoplasm and translation. **(B)** (1)IGF2BP2 promotes the development of OSCC by enhancing the stability of SLC7A11 mRNA. (2) Hoxb-as3 interacts with IGF2BP2 to promote the stability of C-MYC, thereby promoting the proliferation and viability of OSCC cells. (3) Cure GHG manipulates IGF2BP3 through miR-142-35p, thus accelerating the progression of oral squamous cell carcinoma. Epithelial-mesenchymal transition was the main mechanism through which girlGHG/IGF2RP3 promotes metastasis of OSCC. (4) IGF2B3P3 binds to the PDPN mRNA to promote cell migration. (5) CirclCD2 can inhibit proliferation, migration and invasion of OSCC cells and increase cell apoptosis by regulating miR-149-5p/IGFBPI axis (6) Linco1305 mabilizes HTR3A mRNA through interacting with IGF2BP2 and IGF2BP3 and thereby promotes metastasis and proliferation of ESCC (7) RIMIS facilitates laryngeal squamous cell carcinoma progression by regulating TMBIM6 stability through IGF2BP3 dependent. (8) CCAT2 binds to miR-2006 and reduces its expression, leading to up-regulation of IGF2BP2, which enhances the stability of TKI mRNA and its expression by recognizing meA modification of TK1. (9) MiR-454-3p targets IGF2BPI through ERK and AKT signaling pathways and inhibits the proliferation, migration and apoptosis of ESCA cells. (10) IGF2BPI and UHRF2 promote ESCC invasion and proliferation, and inhibit apoptosis through miR-98-5p mediation (11) IGF2BP3 is positively correlated with: I INC00460, which promotes the progress of TSCC through LINC00460/miR-320b/IGF2RP3 axis (12) IGFBP2 may promote the development of hvnoonharyngeal cancer mediated by the PD-1/PD-L1 axis.

In 2018, Huilin Huang and colleagues further explored the role of IGF2BPs family as m6A readers, which uncovered their role in mRNA stability and translation, and confirmed that IGF2BPs can directly bind to MYC CRD and promote MYC expression in a m6A dependent manner (15) (**Figure 2A**). Since then, IGF2BPs has been known as m6A reader. Douglas Hanniford et al. found that IGF2BP3 interacts with CDR1as to mediate the invasion of melanoma induced by CDR1as silencing (16), demonstrating that IGF2BP3 is an important mediator in melanoma development. IGF2BP3 expression is higher in primary renal cell tumors renal metastatic tumors (17). However the specific mechanism is still unclear. Overexpressed IGF2BP3 promotes the proliferation, invasion and metastasis of thyroid cancer by activating PI3K and MAPK pathways (18). The expression of IGF2BP3 is closely related with carcinoembryonic protein—HMGA2in hepatoma cells, which suggesting that IGF2BP3 may be a key factor in the regulation of HMGA2 (19). IGF2BP3 delays the mRNA attenuation of MEK1/ERK pathway through direct interaction with MEKKI, promoting the progression of colorectal cancer (20). Pengpeng Zhu et al. identified a hypoxia-induced lncRNA KB-1980E6.3, which is abnormally upregulated in clinical breast cancer tissues. Upregulated lncRNA KB1980E6.3 forms lncRNA KB-1980E6.3/IGF2BP1/C-MYC signal axis by absorbing IGF2BP1, which maintains the stability of C-MYC mRNA and leads to poor prognosis of breast cancer (21). In one study, a new circRNA, circXPO1, interacts with IGF2BP1 and enhance the stability of β-catenin mRNA, thereby promoting the progression of lung adenocarcinoma (LUAD) (22). Xiaoge Hu et al. found that IGF2BP2 regulates lncRNA DANCR and promote the pathogenesis of pancreatic cancer (23). Many studies have shown that IGF2BPs plays a role in promoting tumor progression in most malignancies, and we are looking forward to exploring its role in head and neck squamous cell carcinoma.

## Expression and role of IGF2BPs in HNSCC

Head and neck squamous cell carcinoma (HNSCC) is the sixth most common cancer worldwide, with about 700,000 cases diagnosed every year (24). Most head and neck tumors arise from mucosal epithelial cells of the mouth, pharynx, and larynx and are collectively known as HNSCC. Oral and throat cancers are often associated with smoking, alcohol abuse, or both, while pharyngeal cancers are increasingly attributed to human papillomavirus (HPV) infection, primarily HPV-16 (25). Due to the lack of specific early diagnosis, HNSCC is usually detected at an advanced stage, with hypopharyngeal squamous cell carcinoma having the worst prognosis. At present, clinical diagnosis of HNSCC is mainly made through clinical manifestations and electronic laryngoscopy and biopsy pathology is the gold standard for diagnosis. There has been a lack of early diagnostic criteria for HNSCC, which is also the main reason for the late diagnosis and poor prognosis of HNSCC. It is particularly urgent to understand the important molecular mechanism of the occurrence and development of HNSCC and find the evidence to judge the occurrence of HNSCC from the gene level by molecular means, so as to achieve the purpose of early detection, early diagnosis and early treatment. IGF2BPs was reported to be closely correlated with HNSCC, which will be summarized as follows. In studies, IGF2BP3 was expressed in patients with stages of HNSCC (pT1-4) and nodule stage (pN0-3), and the expression was statistically significant. In oral cancer patients, IGF2BP3 expression was significantly associated with overall survival (P = 0.038) based on tumor location. Multivariate analysis showed that IGF2BP3 could be used as an independent predictor of oral squamous cell carcinoma (26).

### Oral squamous cell carcinoma

Oral squamous cell carcinoma (OSCC) is the most common oral malignant tumor, and the 5-year survival rate is only 50% (27). Due to the lack of symptoms and lack of awareness of risk factors such as smoking and alcohol, the diagnosis is often late. Chiao-ying Lin, Shengjin Li, and Ki-Yeol Kim’s team found that IGF2BP3 overexpression in OSCC cells was associated with higher histological grade, lymph node metastasis, advanced tumor, and clinical stage. Multivariate analysis showed that IGF2BP3 expression was an independent prognostic indicator of OSCC (28–30. It has been reported that circ-IGHG manipulates IGF2BP3 through miR-142-5p in OSCC, thus accelerating the progression of oral squamous cell carcinoma (31). In tongue squamous cell carcinoma (TSCC) studies, LINC00460 acts as a miRNA molecular sponge to inhibit miR-320b. IGF2BP3 is a potential target of miR-320b, and the expression of IGF2BP3 is positively correlated with LINC00460, which promotes the progress of TSCC through LINC00460/miR-320b/IGF2BP3 axis (32). It has been suggested that IGF2BP3 binds to the 3’UTR of PDPN mRNA, which leads to stabilization of PDPN mRNA. PDPN may be a non-EMT-dependent invasion factor that binds to CD44 to promote targeted cell migration (33). An interesting finding is that EGF stimulation can induce the expression of IGF2BP3 and PDPN. Lymph node metastasis in patients with squamous cell carcinoma is correlated with IGF2BP3 and PDPN expression (34). Young Sun Hwang et al. explored the relationship between IGF2BP3, PDPN and OSCC with bone invasion. Results The expression of IGF2BP3 and PDPN was significantly correlated with T stage, lymph node metastasis and overall survival in OSCC patients. In OSCC patients, dual expression of IGF2BP3 and PDPN was associated with bone infiltration and osteoclast number, while single expression was not. In addition, loss of IGF2BP3 or PDPN inhibits the expression of interleukin (IL-6) and IL-8 in OSCC cells and decreases the expression of NF-KB ligand receptor activator in OSCC xenograft tumor tissues (35). Achille Tarsitano’s team proposed IGF2BP3 and laminin-5 as preoperative predictors of Perineural Invasion (PNI). In their study, 84.8% of PNI positive patients had IGF2BP3 positive expression. The relationship between IGF2BP3 and PNI was statistically significant. Immunohistochemical expression of Laminin-5 in preoperative biopsy materials is significantly correlated with PNI in surgical specimens (36). Lehong Qiu et al. found that down-regulation of circBICD2 could inhibit proliferation, migration and invasion of OSCC cells and increase cell apoptosis by regulating miR-149-5p/IGF2BP1 axis (37). In terms of treatment, Fei Xie et al. verified that IGF2BP1 was significantly related to cisplatin resistance in oral squamous cell carcinoma by establishing cisplatin resistant cell line HN30/DDP. IGF2BP1 can promote cisplatin resistance in oral squamous cells by activating downstream Akt signaling pathway (38). In addition, IGF2BP2 is highly expressed in OSCC and is associated with poor overall survival in OSCC patients. GSEA results showed that apoptosis -, tumor - and immune-related pathways were significantly enriched in IGF2BP2 highly expressed samples. In addition, GO and KEGG enrichment analysis of IGF2BP2 co-expressed genes showed that these genes were mainly related to immunity/inflammation and tumorigenesis (39). It has been found that IGF2BP2 interacts with other RBPs to promote OSCC development. For example, METTL3 enhances the stability of SLC7A11 mRNA through m6A-mediated IGF2BP2 binding, thus contributing to OSCC progression (40). Recent studies have shown that HOXB-AS3 is upregulated in OSCC tissues and is associated with poor prognosis of OSCC. Hoxb-as3 was also found to encode a protein that interacts directly with IGF2BP2 and promotes the stability of C-MYC, thereby promoting the proliferation and viability of OSCC cells (41).Compared with normal tissues, IGF2BP2-AS1 expression is higher in OSCC, which is associated with poor survival in OSCC patients. Down-regulated IGF2BP2-AS1 inhibits cell growth and migration through the Wnt/β-catenin pathway, which may provides a new molecular target for OSCC (42). There are many studies on the molecular mechanism of IGF2BPs in OSCC. In general, the up-regulation of IGF2BPs plays a role in promoting the occurrence and development of OSCC.

### Laryngeal squamous cell carcinoma

Laryngeal carcinoma (LSCC) is one of the most common head and neck malignancies. Despite the continuous improvement of treatment methods, the 5-year survival rate of laryngeal cancer is still unsatisfactory. Understanding the molecular mechanism of laryngeal cancer is very important for the diagnosis and treatment of laryngeal cancer. In previous literature reports, immunohistochemical analysis found that the expression of IGF2BP3 was higher in laryngeal cancer tissues than in tissue adjacent to carcinoma (43). Similarly, Diana Marž I ć et al. first analyzed immunohistochemistry Ki-67 and IGF2BP3 were consistent in the control group and precancerous lesions, but significantly different in LSCC (44). Subsequent studies found that IMP3 expression was lower in benign laryngeal lesions and dysplasia, while diffuse and strong cytoplasmic staining was observed in LSCC (45). All these results support the hypothesis that IGF2BP3 can be used as a new biomarker in the diagnosis of laryngeal cancer. Recent studies was reported that RBM15 mediates the methylation enrichment of TMBIM6 mRNA, and IGF2BP3 can enhance the stability of TMBIM6 (46). IGF2BP3 indirectly promotes the invasion and migration of laryngeal cancer in the process of IGF2BP3-dependent methylation modification. There are few studies on IGF2BP3 in laryngeal cancer, but the existing literature has shown that IGF2BP3 expression is elevated in laryngeal cancer tissue, suggesting that IGF2BP3 plays an important role in the occurrence and development of laryngeal cancer.

### Hypopharyngeal squamous cell carcinoma

The location of hypopharyngeal squamous cell carcinoma (HSCC) is insidious, the early symptoms are non-specific, and its pathology is characterized by easy submucosal spread and local lymph node metastasis (47). It was reported that the incidence and mortality of hypopharyngeal cancer accounted for 4% of all cancer diseases worldwide in 2020 (48), and the prognosis was poor (49). However, the molecular mechanism of hypopharyngeal cancer is very limited. Studies have shown that down-regulation of IGF2BP2 reduces the expression of PD-1/PD-L1, while the presence of PD-L1 inhibitors can reduce the expression of IGF2BP2. Therefore, it is speculated that IGF2BP2 may promote the development of hypopharyngeal cancer mediated by the PD-1/PD-L1 axis (50).

### Esophageal squamous cell carcinoma

Esophageal squamous cell carcinoma (ESCC) is the eighth most common type of cancer worldwide and the sixth leading cause of cancer-related mortality. The survival of ESCC was about 95% at 5 years follow up (51). Esophageal cancers are divided into esophageal adenocarcinoma and esophageal squamous cell carcinoma according to pathological types. ESCC accounts for 90% of esophageal cancers, and ESCC is one of the most aggressive human squamous cell carcinomas. Cervical esophageal carcinoma (CEC) is of great significance to the translational study of CEC because of its high location, which may cause obvious dysphagia and other symptoms in the early stage, and its close proximity to the larynx and other structures makes it difficult to surgery. A study by Huaying Zhao, Wei Guo and Wakita A showed that advanced ESCC patients with high levels of IGF2BP3 expression had A significantly worse prognosis than patients with low levels of IGF2BP3 expression, and IGF2BP3 expression status is an important independent prognosisative factor for patients undergoing surgical treatment alone (52–54). In addition, IGF2BP3 status may be a clinically useful marker for assessing the need for postoperative adjuvant chemotherapy. LINC01305 promotes stability of HTR3A mRNA by interacting with IGF2BP2 and IGF2BP3. In order to describe the function of HTR3A, the authors conducted transwell analysis and proved that HTR3A promotes the migration and proliferation of ESCC (55). Qian LX et al. confirmed that IGF2BP3 affects the stability of KIF18A mRNA through RNA stability experiment. Further studies showed that KIF18A was associated with proliferation, migration, invasion and radiation resistance of esophageal carcinoma cells. IGF2BP3 regulates the progression of esophageal cancer by affecting the mRNA stability of KIF18A in EC cell lines (56).The protein and gene expression levels of miR-454-3p in ESCC tissues and cells were down-regulated compared with those in adjacent normal tissues and normal esophageal epithelial cells. In the mouse subcutaneous transplanted tumor model, miR-454-3p controls IGF2BP1 through ERK and AKT signaling pathways and inhibits the proliferation, migration and apoptosis of ESCA cells. These results suggest that miR-454-3p plays an important role in ESCA by targeting IGF2BP1 (57). Xian-yun Fang et al. found that IGF2BP1 and UHRF2 promote ESCC invasion and proliferation, and inhibit apoptosis through miR-98-5p mediation (58). Xiaodan Wu et al. observed upregulation of CCAT2, IGF2BP2 and TK1 and inhibition of miR-200b expression in ESCC cells and tissues. Further studies demonstrated that CCAT2 binds to miR-200b and reduces its expression, leading to up-regulation of IGF2BP2 expression. IGF2BP2 enhances the stability of TK1 mRNA and enhances its expression by recognizing m6A modification of TK1. CCAT2 promotes migration and invasion of ESCC cells by up-regulating TK1 expression *in vivo* and *in vitro (* 59), (**Figure 2B**).

## Significance of IGF2BPs in the treatment of HNSCC

IGF2BP3 is significantly elevated in HNSCC and is closely related to its occurrence and development. IGF2BP3 is a potential target for the treatment of HNSCC, but there is no specific targeted therapy at present. Studies have shown that the expression of IGF2BP3 in radiation-resistant TE-5 and TE-9 cells is higher than that in radiation-sensitive TE-12 clones. When the expression of IGF2BP3 in TE-5 and TE-9 cells is knocked out by siRNA, their sensitivity to radiation is enhanced (60). This also fully proves that IGF2BP3 has a profound effect on treatment. Le Xu et al. reported that IGF2BP2 stabilized METTL3 methylation promoting OSCC, while triptolide inhibited OSCC by inhibiting METTL3-SLC7A11 axis. So triptolide has the potential to be effective anti-OSCC agents targeting METTL3 (40). It has been suggested that RBM39 is a key link in the RBP network in acute myeloid leukemia (AML), and deletion of RBM39 provides a therapeutic strategy for AML with RBP splicing mutations. A class of compounds known as anticancer sulfonamides can selectively degrade RBM39 (61). HuR has been found to be abnormally expressed in a variety of tumors, and the molecular inhibitor MS-444 was first discovered by Meisner et al. in 2007 (62). Targeted immunotherapy for RBP network, especially for IGF2BP3 related targets, will bring new good news for HNSCC treatment.

## Research prospect of IGF2BPs

IGF2BPs has been rarely studied in head and neck squamous cell carcinoma, but its function in malignant tumors is selfevident. Understanding the molecular mechanism of IGF2BPs and finding the target of IGF2BP3 will open a new direction for the treatment of HNSCC. Meanwhile, IGF2BPs can be used as a novel molecular marker to improve the early diagnosis of HNSCC. Hypopharyngeal cancer is relatively rare in HNSCC, accounting for about 3% of head and neck cancers, but hypopharyngeal cancer is one of the tumors with the worst prognosis among all head and neck cancers, with a reported 5-year overall survival rate of about 30-35% (63). Currently, the expression and related functional mechanisms of IGF2BP3 in hypopharyngeal cancer have not been reported, and the study of IGF2BP3 in hypopharyngeal cancer may bring an important breakthrough to solve the bottleneck of clinical diagnosis and treatment.

## Conclusion

IGF2BPs was discovered as a fetal growth factor. At first, people’s understanding of IGF2BPs was limited to its widespread and high expression in embryos and tumor tissues, and IGF2BP2 is even closely related to metabolic diseases. A large number of studies on the expression enhancement or *de novo* synthesis of IGF2BPs in human cancers and animal models strongly support the important role of IGF2BPs in the control of embryonic development and its role as a carcinogen in most cancers. With the recognition of IGF2BPs’ role in the m6A process, more attention has been paid to the study of IGF2BPs binding to RNA and maintaining its stability. The role of IGF2BPs in promoting cancer has also been found to be closely related to its role as m6A reader. More and better evidence is needed to prove this. Whether the role of IGF2BPs in promoting growth and metabolism is also related to its role as a member of RBPs and what role it plays in the process of growth and metabolism has not been clearly reported. Whether IGF2BPs has a unified function to regulate various life activities of the human body still needs further research.

The structural domain of IGF2BPs is highly similar, which is related to its similar function. We know that both KH and RRM domains contribute to RNA binding. Although the KH domain plays a central role in many pathophysiological processes in prokaryotes and eukaryotes, little is known about the biophysical properties and folding dynamics of the domain. In this case, the study of folding is helpful to understand the folding properties of KH domain and identify the existence of metastable intermediate states. Whether KH mechanism domain has other functions, such as related to amyloid fiber formation (64). Further study of the domain is needed to explore more hidden functions of IGF2BPs.

A large number of literatures have proved that IGF2BPs is related to tumor progression, but there is still no design for IGF2BPs-related inhibitors, and this targeted immunotherapy is the best approach for precise treatment. Head and neck squamous cell carcinoma is often located in the throat tract with complex anatomical structure. Surgery greatly affects the quality of life of patients, and the effect of radiotherapy and chemotherapy is not very rational. Therefore, it is urgent to intervene with drugs targeting IGF2BPs to achieve clinical transformation effect.

## Author contributions

KW and WL retrieved concerned literatures and wrote the manuscript. TS designed the figures. DL and FC revised the manuscript. All authors contributed to the article and approved the submitted version.

## Conflict of interest

The authors declare that the research was conducted in the absence of any commercial or financial relationships that could be construed as a potential conflict of interest.

## Publisher’s note

All claims expressed in this article are solely those of the authors and do not necessarily represent those of their affiliated organizations, or those of the publisher, the editors and the reviewers. Any product that may be evaluated in this article, or claim that may be made by its manufacturer, is not guaranteed or endorsed by the publisher.
